# Toward a Clinical Consumer Psychology

**DOI:** 10.3389/fpsyg.2022.904843

**Published:** 2022-06-23

**Authors:** Steven S. Posavac, Heidi D. Posavac, Donald R. Gaffney, Frank R. Kardes

**Affiliations:** ^1^Owen Graduate School of Management, Vanderbilt University, Nashville, TN, United States; ^2^Private Practitioner, Nashville, TN, United States; ^3^Lindner College of Business, University of Cincinnati, Cincinnati, OH, United States

**Keywords:** clinical psychology, consumer welfare, judgment and decision making, marketing, social cognition

## Abstract

This article promotes the development of clinical consumer psychology; the study of how dysfunctional and maladaptive cognitive and behavioral processes interact with individuals’ consumer experience and behaviors. The article is organized around three primary discussion points: (a) A definition of clinical consumer psychology, supported by illustrative examples of recent research. (b) The delineation of 10 broad priorities for future work that can be used to generate specific research possibilities. (c) How the field will benefit if researchers work within the clinical consumer psychology paradigm, and the bi-directional relationship whereby research in this vein would benefit both fields in which judgment and decision processes are focal (e.g., consumer psychology, marketing, and social cognition) as well as clinical psychology.

## Introduction

Individuals have a constellation of experiences, tendencies, and genetics that affect their perceptions and thoughts, as well as guide behavior and choice. Unfortunately, individuals sometimes exhibit dysfunctional and maladaptive cognitions and behaviors that rise to the level of clinical concern. This article reviews some recent research at the interface of clinical psychology and consumer behavior to demonstrate how work on clinical consumer psychology can deepen our understanding of consumption, as well as the human experience more generally. Despite the potential conceptual and practical value of research at this nexus, extant work is sparse. Accordingly, we offer suggestions for future research in the hopes of sparking interest in what we hope will become a growing psychological subfield.

A starting point for clinical consumer psychology is understanding how a given clinical disorder may manifest in consumer judgments and choices. Such research would deepen both our conceptions of the disorder, as well as consumption behavior itself. Moreover, learning how clinical symptoms can be exacerbated or ameliorated by consumption behaviors also has significant promise, as does understanding the role of marketing communications themselves in clinical phenomena.

## Illustrative Recent Work in Clinical Consumer Psychology

Although we can only cover a few clinical psychology topics here, we hope that defining the clinical consumer psychology gestalt through examples of recent research will encourage future work. The clinical conditions we discuss are described in the *Diagnostic and Statistical Manual of Mental Disorders* (DSM-5; American Psychiatric Association, 2013). Generally, if a condition is included in the DSM-5, the interplay or relation between it and an individual’s behavior in the marketplace would fall under our conceptual umbrella, as would marketing activity that contributes to its development or maintenance. In contrast, sub-clinical phenomena, for example “retail therapy” to improve a transitory mood (Rick et al., 2014), would not be a clinical consumer psychology topic.

The following examples are illustrative of the value of the study of clinical consumer psychology versus exhaustive. Together they reflect the breadth of clinical phenomena and consumer behaviors that may be fruitful for researchers to explore.

### Anxiety Disorders

Research in clinical psychology typically considers the causes, consequences, prevention, and treatment of maladies. Posavac and Posavac (2017) proposed that clinical phenomena may be also associated with vulnerability to specific types of persuasive marketing attempts. Although there are multiple variants of anxiety disorders, one commonality is that individuals in an anxious state are motivated to seek amelioration of their distress. Accordingly, marketing themes that may help to reduce the source of anxiety may be received differentially favorably.

Posavac and Posavac (2017) focused on adult separation anxiety disorder (ASAD): the excessive fear of being separated from those with whom one is attached. Examples of ASAD symptoms include distress when separation from home or attachment figures is imagined or occurs, worry about something happening that would lead to losing or being separated from an attachment figure, and fear regarding being away from home. In Posavac and Posavac’s (2017) study ASAD symptomology was measured, then following several distractor items, participants reviewed an advertisement that either contained or did not contain a “coming home” appeal, and then rated their advertisement attitudes. Individuals high in ASAD symptomology were particularly susceptible to the advertisement when it appealed to home, but those with no and lower symptomology were not. In addition to insight regarding ASAD, this study shows that clinically relevant phenomena can affect individuals’ responses to marketing.

### Stressor-Related Disorders

Russell et al. (2019) explored the relation between stressors from combat and deployment experiences among military members and consumption choices when a deployment ends. Deployment stressors such as being away from home and the unavailability of commodities were associated with more materialism and compulsive buying, but combat stressors, including being shot at and being exposed to carnage during a war-time engagement, demonstrated the opposite pattern. Furthermore, Russell et al. (2019) reported that stressors increase the likelihood of maladaptive consumption (e.g., substance abuse) by making mental health outcomes such as post-traumatic stress disorder, anxiety, or depression worse. Thus, they helped to fill a conceptual gap by delineating new drivers of maladaptive consumer behavior, and more generally demonstrated that clinically relevant experiences can affect choice.

### Personality Disorders

Clinical psychological phenomena have also been shown to affect consumer judgment and decision making. Posavac et al. (2022) explored how personality disorder symptoms relate to consumer processes including attribute weighting, product feature tradeoffs, choice, and willingness to pay. Posavac et al. (2022) focused on histrionic personality disorder (HPD), which is characterized by shallow yet overly dramatic emotionality, a strong desire for attention, and inappropriately seductive behavior. Higher HPD symptomology was related to consumers placing more weight on attributes that enable a product to capture attention for its owner, the probability of choice of products that have strong attention-grabbing potential, trading off tangible and desirable attributes central to core product function to obtain a product better able to put the spotlight on the owner, and being more willing to pay for products with high attention attracting capacity. Although these HPD insights are valuable, the documentation of how clinical phenomena can affect consumer judgment and decision making processes is of broader importance.

### Addictive Disorders

The foregoing examples demonstrate how clinical phenomena can impact responses to marketing and processes that drive consumer judgment and choice. Martin et al. (2013) took the converse perspective and explored how marketing messaging can impact individuals in a clinically relevant way. Specifically, they described a model of addiction and how marketing cues can affect, positively or negatively, whether individuals become addicted to a product (e.g., drugs, alcohol) or service (e.g., gambling). Martin et al. (2013) delineated four stages along a continuum: non-use of a product or service, non-addictive use, near-addictive use, and addiction. Moreover, they offered examples of how marketing can move individuals toward or away from addiction. Thus, in addition to offering deeper insight into an important clinically relevant phenomenon by considering the role of marketing, they were also able to identify ways in which marketing can affect individuals’ psychological outcomes.

Pathological gambling is a particularly nefarious behavioral addiction from a social welfare perspective because it has such destructive consequences for people’s lives (American Psychiatric Association, 2013), and opportunities to gamble are becoming more readily available (Jones, 2021). Gambling disorder is characterized by recurring uncontrollable gambling that disrupts one’s personal, family, and work experience. Interestingly, there are strong similarities between substance addition and gambling disorder (Leeman and Potenza, 2012) predicated on overlapping neurocognitive drivers of each addiction (Brevers et al., 2013). This research, similar to work on compulsive buying behavior (Granero et al., 2016), underscores the importance of investigating everyday consumer decision making from the perspective of clinical neuroscience and neuropsychology (see also Clark et al., 2013).

### Body Image and Eating Disorders

A large body of research explores the role of media images of attractiveness on women’s body image, as well as the likelihood of disordered eating behavior (see Levine and Harrison, 2009 for a review). The media ideal for female attractiveness is unrealistically attractive, with an emphasis on thinness. Given the financial incentive for firms to present an ideal that is discrepant from the self-perceptions of the vast majority of females, and then sell mechanisms to ostensibly refine one’s appearance and thereby reduce the discrepancy (Mandel et al., 2017), unhealthy body-related messaging predominates. Consequently, marketing can psychologically harm individuals.

## Promising Directions in Clinical Consumer Psychology

Although clinical consumer psychology research to date is nascent, it has strong potential to add conceptual and practical value. We next pose 10 questions that we believe are fruitful directions for a new science of clinical consumer psychology. The questions are framed generally, and myriad research possibilities can be derived from each.

### How Do Clinical Constructs Drive Individuals’ Responses to Marketing?

Specific directions include investigating what is persuasive or motivating for individuals with a given diagnosis or set of symptoms, and if particular types of communications are sought or avoided. Generally, communications that speak to a motivation, a need, or a concern will be compelling, whereas marketing that activates distress may be avoided or be of low effectiveness.

### What Are the Roles of Clinical Constructs in Consumer Behavior Processes?

As Posavac et al. (2022) demonstrated, clinical phenomena may have a role in driving or moderating a host of processes relevant to judgment and choice. Moreover, there may be systematic differences in what is purchased and how (O’Guinn and Faber, 1989) as a function of clinical symptomology. For example, how are options generated for consideration (Thomas et al., 2014), how are alternatives compared, and which option is ultimately chosen? Research in this vein has tremendous promise for deepening our understanding of both clinical conditions as well as consumption behavior.

### How Can Marketers Leverage Clinical Phenomena to Drive Profitability?

Although rife with ethical issues, understanding consumer motivation through a clinical lens may lead to greater precision in segmentation and targeting, and profitability. For example, marketers have long targeted over-the-counter drug communications at hypochondriacs (Berkowitz, 2017). There may be many clinical phenomena that correlate with the proclivity to consider and purchase certain goods and services, and that thus may be used as bases for consumer segmentation.

A converse possibility is that marketers may use clinical consumer psychology knowledge to avoid targeting vulnerable segments. Such a strategy would be ethically sound, and likely to be fiscally wise as well due to potential public relations benefits of responsible corporate behavior and avoiding the ire of regulatory agencies.

### How Do Individuals Use Consumption to Alleviate Distress? Conversely, Can Consumption Exacerbate Clinical Symptoms?

When distress is experienced along a clinical dimension, consumers may use consumption in a variety of ways to ameliorate this distress. Research in this area would start by understanding the nature of distress that individuals with a given diagnosis experience, then considering how choice and consumption of products and services can reduce felt distress.

Unfortunately, consumption may sometimes reinforce, maintain, or exacerbate clinical symptoms. For example, women with restrained eating tendencies are likely to be shown pro-anorexic material when consuming social media (Gerrard, 2020) due to the recommendation algorithms that match users’ prior information consumption with suggested content. An important line of inquiry for clinical consumer psychology going forward will be to identify when and how consumption of products, services, and information can make clinically relevant inclinations or full-blown disorders more intransient or of worse intensity.

### Are There Associations Between Clinical Phenomena and Consumer Decision-Making Tendencies?

Interesting foci include whether the magnitude of certain tendencies in consumer judgment and choice covary with the presence or extent of clinical phenomena. For example, Sanchez and Dunning (2021) demonstrated that the tendency to jump to conclusions, an important driver of maladaptive cognitive processing among schizophrenics, is associated with errors in reasoning, holding false beliefs, and overconfidence. It is likely that other clinical phenomena are related to tendencies documented in the large judgment and decision making literature (Hastie and Dawes, 2010).

### How, When, and Why Does Marketing Adversely Affect Individuals?

Research that documents ways in which marketing can cause consumers psychological harm can be very important both from the perspective of marketing ethics, as well as understanding the specifics of the harm that may be done. A corollary goal is identifying who is at most risk, and why, because such knowledge may be useful in facilitating mental health (Posavac and Posavac, 2020).

### How Can Consumer Psychology Concepts Contribute to Clinical Psychological Interventions?

Research in this area may be less common because consumer psychologists are generally not well versed in clinical treatments. However, Russell et al. (2019) and Posavac et al. (2001) have created and tested interventions using media literacy and psychoeducational approaches to prevent youth alcohol abuse and body image disturbance in women, respectively. The key to the development of both of these interventions was understanding the underlying social cognitive processes that drive each malady.

### When Does Marketing Itself Make an Extant Condition Better?

Marketing can contribute to the knowledge of how to reduce important self-discrepancies, treat given maladies, and the simple act of learning that options exist may alleviate distress when consumers learn that others share their condition. For example, direct-to-consumer messaging and prescription discount cards can raise awareness of mental health conditions as well as remedies. It would be valuable to learn more about when and how such marketing activity may be beneficial for individuals.

### How Does Physical Harm to the Brain Affect Consumption?

What are the consumer consequences of traumatic brain injuries, strokes, mild cognitive impairments, and so on? These questions will be of growing importance given the incidence of war-related head injuries, a growing appreciation of the incidence of chronic traumatic encephalopathy in sport, and an aging population.

### Can Consumption Behavior Be Used to Improve Diagnostic Accuracy, and Track Treatment Progress?

As the study of clinical consumer psychology advances, the accumulated learnings may have value for the treatment of disorders. Specifically, when patterns in consumption behaviors emerge among individuals with extant diagnoses, clinicians observing such behaviors during assessment of new patients may be able to develop more nuanced profiles, potentially aiding diagnostic accuracy, and particularly differential diagnosis. Moreover, when given consumption behaviors result from a clinical condition, decreases in the frequency or extremity of such behaviors may be a signal of the efficacy of ongoing treatment, and may themselves sometimes be a target of therapy.

## Conclusion

It is our hope that defining the clinical consumer psychology paradigm and delineating a set of questions for future research will lead readers to share our excitement for the conceptual and social value that work in this space may have. Indeed, developing a robust clinical consumer psychology literature has strong potential to both deepen our understanding of the human experience, as well as to improve it.

## Author Contributions

SP wrote the manuscript. All authors contributed to the conceptual development of the manuscript, and read and approved the submitted version.

## Funding

All funding for this paper was provided by Vanderbilt University through SP’s research account.

## Conflict of Interest

The authors declare that the research was conducted in the absence of any commercial or financial relationships that could be construed as a potential conflict of interest.

## Publisher’s Note

All claims expressed in this article are solely those of the authors and do not necessarily represent those of their affiliated organizations, or those of the publisher, the editors and the reviewers. Any product that may be evaluated in this article, or claim that may be made by its manufacturer, is not guaranteed or endorsed by the publisher.

## References

[ref1] American Psychiatric Association (2013). Diagnostic and Statistical Manual of mental Disorders. 5th Edn. Washington, DC: American Psychiatric Association.

[ref2] Berkowitz E. N. (2017). Essentials of Healthcare Marketing 4th Edn. Burlington, MA: Jones & Bartlett Learning.

[ref3] Brevers D. Bechara A. Cleeremans A. Noel X. (2013). Iowa gambling task (IGT): twenty years after – gambling disorder and IGT. Front. Psychol. 4:665. doi: 10.3389/fpsyg.2013.00665 24137138 PMC3786255

[ref4] Clark L. Averbeck B. Payer D. Sescousse G. Winstanley C. A. Xue G. (2013). Pathological choice: the neuroscience of gambling and gambling addiction. J. Neurosci. 33, 17617–17623. doi: 10.1523/JNEUROSCI.3231-13.2013, PMID: 24198353 PMC3858640

[ref5] Gerrard Y. (2020). TikTok has a pro-anorexia problem. Wired. Available at: https://www.wired.com/story/opinion-tiktok-has-a-pro-anorexia-problem/ (Accessed May 18, 2022).

[ref6] Granero R. Fernández-Aranda F. Mestre-Bach G. Steward T. Baño M. del Pino-Gutiérrez A.. (2016). Compulsive buying behavior: clinical comparison with other behavioral addictions. Front. Psychol. 7:914. doi: 10.3389/fpsyg.2016.00914 27378999 PMC4908125

[ref7] Hastie R. Dawes R. M. (2010). Rational Choice in an Uncertain World: The Psychology of Judgment and Decision Making 2nd Edn. Thousand Oaks, CA: Sage Publications, Inc.

[ref8] Jones Z. (2021). Rise of the IGaming industry: Is the United States ready to accept online casinos? Forbes. Available at: https://www.forbes.com/sites/zackjones/2021/04/21/the-rise-of-the-igaming-industry-what-is-in-store-for-the-citizens-of-united-states/?sh=796fb8863555 (Accessed May 18, 2022).

[ref9] Leeman F. L. Potenza M. N. (2012). Similarities and differences between gambling disorder and substance use disorders: a focus on impulsivity and compulsivity. Psychopharmacology 219, 469–490. doi: 10.1007/s00213-011-2550-7, PMID: 22057662 PMC3249521

[ref10] Levine M. P. Harrison K. (2009). “Effects of media on eating disorders and body image,” in Media Effects: Advances in Theory and Research. 3rd Edn. eds. Bryant J. Oliver M. B. (New York: Routledge), 490–515.

[ref11] Mandel N. Rucker D. D. Levav J. Galinsky A. D. (2017). The compensatory consumer behavior model: how self-discrepancies drive consumer behavior. J. Consum. Psychol. 27, 133–146. doi: 10.1016/j.jcps.2016.05.003

[ref12] Martin I. M. Kamins M. A. Pirouz D. M. Davis S. W. Haws K. L. Mirabito A. M.. (2013). On the road to addiction: the facilitative and preventive roles of marketing cues. J. Bus. Res. 66, 1219–1226. doi: 10.1016/j.jbusres.2012.08.015

[ref13] O’Guinn T. C. Faber R. J. (1989). Compulsive buying: a phenomenological exploration. J. Consum. Res. 16, 147–157. doi: 10.1086/209204

[ref14] Posavac H. D. Posavac S. S. Weigel R. G. (2001). Reducing the impact of media images on women at risk for body image disturbance: three targeted interventions. J. Soc. Clin. Psychol. 20, 324–340. doi: 10.1521/jscp.20.3.324.22308

[ref15] Posavac S. S. Kardes F. R. Posavac H. D. Gaffney D. R. (2022). The utility of clinical psychology concepts for judgment and decision-making research: The case of histrionic features. Personal. Soc. Psychol. Bull. 48, 65–77. doi: 10.1177/0146167220980887, PMID: 33514279

[ref16] Posavac S. S. Posavac H. D. (2017). Adult separation anxiety disorder symptomology and susceptibility to marketing persuasion. J. Soc. Clin. Psychol. 36, 158–169. doi: 10.1521/jscp.2017.36.2.158

[ref17] Posavac S. S. Posavac H. D. (2020). Adult separation anxiety disorder symptomology as a risk factor for thin-ideal internalization: The role of self-concept clarity. Psychol. Rep. 123, 674–686. doi: 10.1177/0033294119829440, PMID: 30744502

[ref18] Rick S. I. Pereira B. Brunson K. A. (2014). The benefits of retail therapy: making purchase decisions reduces residual sadness. J. Consum. Psychol. 24, 373–380. doi: 10.1016/j.jcps.2013.12.004

[ref19] Russell C. A. Hamby A. M. Grube J. W. Russell D. W. (2019). When do public health epilogues correct the influence of alcohol story lines on youth? The interplay of narrative transportation and persuasion knowledge. J. Public Policy Mark. 38, 316–331. doi: 10.1177/0743915618818567

[ref20] Russell C. A. Russell D. W. Hill R. P. (2019). “Post-traumatic stress and consumption behaviors,” in Advances in Consumer Research. Vol. 47 eds. Bagchi R. Block L. Lee L. (Duluth, MN: Association for Consumer Research), 142–145.

[ref21] Sanchez C. Dunning D. (2021). Jumping to conclusions: implications for reasoning errors, false belief, knowledge corruption, and impeded learning. J. Pers. Soc. Psychol. 120, 789–815. doi: 10.1037/pspp0000375, PMID: 33252973

[ref22] Thomas R. Dougherty M. R. Buttaccio D. R. (2014). Memory constraints on hypothesis generation and decision making. Curr. Dir. Psychol. Sci. 23, 264–270. doi: 10.1177/0963721414534853

